# Congenital anomalies of the upper digestive tract: a review of esophageal and gastric embryology and malformations

**DOI:** 10.1590/0100-3984.2025.0091

**Published:** 2026-04-23

**Authors:** Bianca Hallage, Gustavo Yano Callado, Raphaela Coelho Veloso Gomes, Rita de Cássia Sanchez, Edward Araujo Júnior, Maria Lúcia de Pinho Apezzato, Romy Schmidt Brock Zacharias, Heron Werner, Eduardo Félix Martins Santana

**Affiliations:** 1 Faculdade Israelita de Ciências da Saúde Albert Einstein (FICSAE), Hospital Israelita Albert Einstein, São Paulo, SP, Brazil.; 2 Department of Obstetrics, Escola Paulista de Medicina - Universidade Federal de São Paulo (EPM-UNIFESP), São Paulo, SP, Brazil.; 3 Department of Fetal Medicine, Biodesign Laboratory DASA/Pontifícia Universidade Católica do Rio de Janeiro (PUC-Rio), Rio de Janeiro, RJ, Brazil.

**Keywords:** Congenital abnormalities, Esophageal atresia, Tracheoesophageal fistula, Pyloric stenosis, hypertrophic, Digestive system abnormalities, Anormalidades congênitas, Atresia esofágica, Fístula traqueoesofágica, estenose pilórica hipertrófica, Anormalidades do sistema digestório

## Abstract

Congenital anomalies of the digestive system, particularly in the esophagus and stomach, present significant challenges to neonatal health due to their complex management and associated risks of morbidity and mortality. This narrative review explores esophageal and gastric anomalies, addressing the embryology, prevalence, diagnostic methods, and treatment approaches, with an emphasis on early detection and improved outcomes. Because of recent advances in prenatal imaging, such as ultrasonography and magnetic resonance, diagnostic accuracy has increased, allowing better clinical planning and management. Studies reveal that gastrointestinal anomalies account for approximately 20% of congenital malformations globally, having a substantial impact on neonatal health and healthcare systems. This review discusses critical anomalies, including esophageal atresia, tracheoesophageal fistula, antral stenosis, and antral atresia, with a focus on diagnostic criteria, surgical interventions, and prognostic factors. By highlighting current knowledge and best practices, we aim to underscore the importance of early, accurate diagnosis and continuous follow-up, which can ultimately improve neonatal outcomes and quality of life.

## Abstract (pt)

As anomalias fetais do sistema digestivo, particularmente no esôfago e no estômago, apresentam desafios significativos para a saúde neonatal devido ao seu tratamento complexo e aos riscos associados de morbidade e mortalidade. Esta revisão narrativa explora a embriologia, a prevalência, os métodos de diagnóstico e as abordagens de tratamento para anomalias esofágicas e gástricas, com ênfase na detecção precoce e na melhoria dos resultados. Utilizando os avanços recentes em imagens pré-natais, como ultrassonografia e ressonância magnética, a precisão do diagnóstico aumentou, permitindo um melhor planejamento clínico e tratamento. Estudos revelam que as anomalias gastrointestinais são responsáveis por aproximadamente 20% das malformações congênitas em todo o mundo, refletindo um impacto substancial na saúde neonatal e nos sistemas de saúde. A revisão discute anomalias críticas, incluindo atresia esofágica, fístula traqueoesofágica e estenose e atresia antral, com foco nos critérios diagnósticos, intervenções cirúrgicas e fatores prognósticos. Ao destacar o conhecimento atual e as melhores práticas, este estudo visa ressaltar a importância do diagnóstico precoce e preciso e do acompanhamento contínuo, contribuindo, em última análise, para melhores resultados neonatais e qualidade de vida.

## INTRODUCTION

Congenital anomalies of the digestive system, particularly in the esophagus and stomach, pose significant neonatal health challenges due to the complexity of their management and the associated morbidity. Although advances in ultrasound and magnetic resonance imaging (MRI) allow intrauterine diagnosis, these anomalies continue to be difficult to manage and require coordinated perinatal care^**(1,^2^)**^. Globally, they constitute approximately 20% of all congenital anomalies; in Brazil, they have a substantial impact on neonatal health, increasing demand for early interventions and intensive care^**(3)**^.

Given the importance of early diagnosis and intervention^**(3)**^, studying esophageal and gastric anomalies can enhance prenatal follow-up and postnatal treatment. This review explores malformations of the upper digestive tract, addressing the embryology, prevalence, prognosis, and emerging therapies. It highlights the need for accurate diagnosis and continuous follow-up to optimize neonatal outcomes and mitigate long-term effects.

This narrative review was conducted through a structured search of the PubMed, Scientific Electronic Library Online, and Latin-American and Caribbean Health Sciences Literature databases, covering the period from database inception to June 2024. The search strategy combined descriptors related to “fetal gastrointestinal malformations,” “esophageal atresia,” and “gastric anomalies,” with emphasis on prenatal imaging and postnatal outcomes. Articles were selected based on clinical relevance, methodological quality, and focus on human studies. Additional references from major society guidelines and radiology reviews were also included.

## EMBRYOLOGY AND DESCRIPTION OF ANOMALIES OF THE GASTROINTESTINAL TRACT

### Overview

During the third and fourth weeks of embryonic development, the endoderm forms the primitive intestinal tube, which differentiates into the foregut, midgut, and hindgut, giving rise to the esophagus, stomach, and intestines^**(1)**^, as detailed in Chart 1. Malformations originate during these early organogenesis stages and can be detected prenatally by ultrasound and advanced imaging techniques^**(2,^3^)**^. Gastrointestinal anomalies account for approximately 20% of all congenital defects^**(4)**^, with anomalies are most common in white male infants, who represent approximately 60% of cases^**(^7^)**^, and are often associated with a positive family history. Among 372 cases evaluated in Brazil^**(5)**^, 44.1% were cases of esophageal atresia with tracheoesophageal fistula, 23.4% were cases of isolated esophageal atresia, 29.3% were cases of congenital esophageal stenosis, and 17.8% were cases of hypertrophic pyloric stenosis. This review focuses on esophageal and gastric anomalies because of their clinical and prognostic relevance.

### Esophagus

#### Developmental aspects

**Figure 1:**
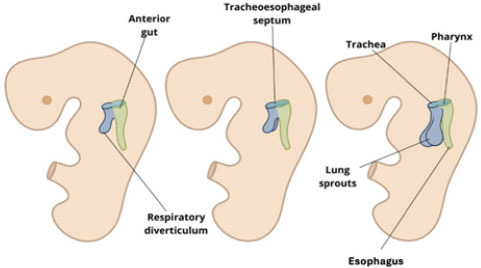
Schematic illustration of embryonic development of the initial digestive and respiratory tracts, created for didactic purposes.

The esophagus develops from the foregut (Chart 1). Initially short, it lengthens as the lungs and heart descend, reaching a proportional length by the seventh week of gestation. The upper two-thirds consist of striated muscle, innervated by the vagus nerve, while the lower third contains smooth muscle, innervated by the splanchnic plexus. The tracheoesophageal septum separates the esophagus from the trachea^**(8)**^, as shown in Figure 1, which also shows the esophagus positioned posterior to the trachea. The absence of content, especially in the first trimester, complicates ultrasound visualization of the esophagus. From week 18, increasing amniotic fluid ingestion and esophageal layer development improve visibility, although identification remains reliant on indirect findings like transient distension during swallowing. In the third trimester (from week 28 onward), esophageal thickening and distension further aid detection; nevertheless, sonographic visualization remains challenging^**(9)**^. The most common esophageal anomalies are esophageal atresia and tracheoesophageal fistula. Although they can occur independently, they are usually seen together. Tracheoesophageal fistula affects the gastrointestinal and respiratory tracts, occurring in ≈ 1 in 3,500–4,500 live births, compared with ≈ 1 in 10,000 for isolated esophageal atresia^**(10–^13^)**^.

**Chart 1:** —The three segments of the gastrointestinal tract and the structures originating from each one.

Segment	Structures originating from each segment
Foregut	Pharynx; esophagus; stomach; liver; gallbladder; pancreas; and cranial portion of the duodenum
Midgut	Caudal portion of the duodenum; jejunum; ileum; ascending colon; and the first two-thirds of the transverse colon
Hindgut	Final third of the transverse colon; descending colon; and rectum

#### Esophageal atresia and tracheoesophageal fistula

##### Definition

Esophageal atresia, the most common esophageal malformation, results from spontaneous posterior deviation of the tracheoesophageal septum (Figure 1) or mechanical displacement of the dorsal foregut. This leads to narrowing or absence of an esophageal segment. In approximately 90% of cases, esophageal atresia is accompanied by a tracheoesophageal fistula, classified by the Gross system^**(14)**^.

**Figure 2:**
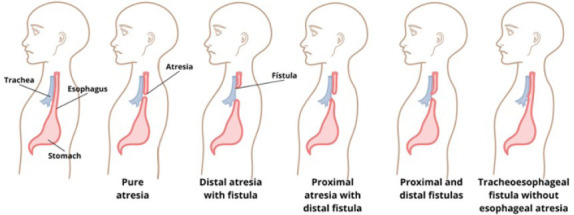
Schematic illustration of the Gross classification, created for didactic purposes.

Imaging is essential for diagnosis. Although ultrasound is widely used because of its accessibility, MRI offers high sensitivity in assessing gastrointestinal anatomy. These modalities also enable anatomical classification^**(15)**^ of esophageal atresia and tracheoesophageal fistula (Figure 2).

Pure esophageal atresia (Gross type A), in which the proximal esophagus ends in a blind pouch without a fistula, occurs in ≈ 7% of cases. In A atresia, there is no passage of liquid or air into the gastrointestinal tract; therefore, the stomach is not visible on ultrasound and newborns present with a scaphoid abdomen. Atresia with a proximal fistula (Gross type B) is rarer, occurring in only ≈ 2% of cases. In this form, the proximal esophagus terminates in a blind pouch but also communicates with the trachea, allowing esophageal fluid to enter the lungs. The stomach remains unfilled and is difficult to visualize. The most common type, seen in ≈ 85% of cases^**(16)**^, is proximal atresia with a distal fistula (Gross type C), in which conventional radiography reveals an aerated stomach and intestinal loops due to communication with the respiratory tract, along with significant gastric distention. A less common variant, occurring in < 1% of cases, is Gross type D, in which there are proximal and distal fistulas. Finally, although tracheoesophageal fistula without esophageal atresia (Gross type E, or H-type fistula) accounts for only ≈ 4% of all cases of esophageal atresia, its diagnosis requires caution because, unlike other forms of atresia, it allows the passage of a nasogastric tube.

##### Diagnosis

During embryonic development, esophageal atresia prevents the passage of amniotic fluid through the intestinal tract, leading to fluid accumulation in the gestational sac in 60–90% of cases^**(1)**^. This condition, known as polyhydramnios, is one of the earliest and most significant prenatal warning signs. The absence of a stomach bubble on ultrasound may suggest this malformation. Studies indicate that a stomach bubble that is small or absent, in conjunction with polyhydramnios, has a sensitivity of 56% as a predictor of esophageal atresia^**(17)**^.

**Figure 3:**
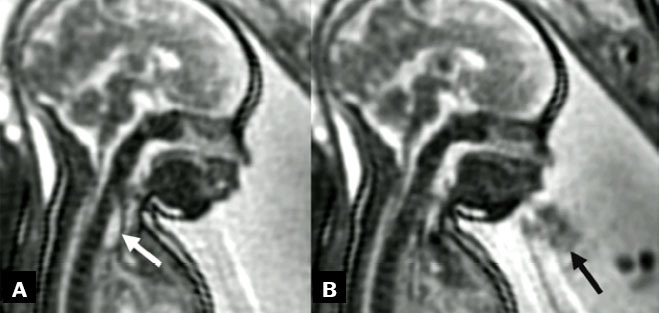
Two-dimensional ultrasound images, acquired with a convex 3.5 MHz transducer in the sagittal and axial planes (**A** and **B**, respectively), of a fetus with esophageal atresia at 33 weeks of gestation. The images show the esophageal pouch filled with fluid (white arrow, in A), followed by fetal regurgitation and an empty esophagus (black arrow, in B).

**Figure 4:**
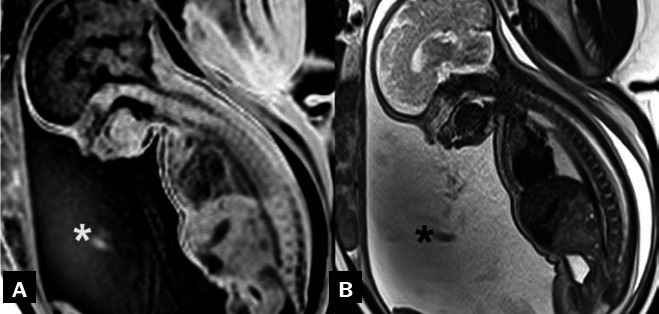
Fetal MRI. Sagittal T1- and T2- weighted sequences (**A** and **B**, respectively), of a fetus with esophageal atresia at 33 weeks of gestation, demonstrating polyhydramnios (asterisks).

**Figure 5:**
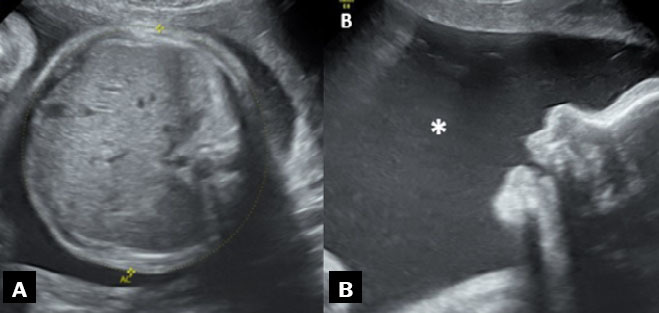
Two-dimensional ultrasound images, acquired with a convex 3.5 MHz transducer in the axial and sagittal planes (**A** and **B**, respectively), of a fetus with esophageal atresia at 30 weeks of gestation, showing the absence of a stomach in the abdomen, together with polyhydramnios (asterisk).

**Figure 6:**
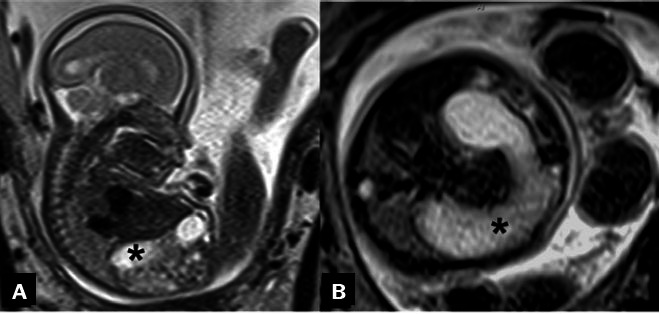
Fetal MRI. Sagittal and axial T2- weighted sequences (**A** and **B**, respectively) obtained at 22 weeks of gestation in a fetus with duodenal and esophageal atresia. The combination of duodenal atresia and esophageal atresia without tracheoesophageal fistula leads to a closed loop of bowel involving the distal esophagus, stomach, and duodenum, visualized prenatally as a characteristic dilated C-shaped fluid collection in the fetal abdomen (asterisks).

Prenatal differential diagnoses include conditions that prevent stomach bubble visualization on imaging and are associated with polyhydramnios. These comprise two main groups of pathologies: those that reduce or alter fetal swallowing of amniotic fluid; and those that increase its production. Consequently, differential diagnoses include gastrointestinal obstructions, urinary tract malformations, congenital infections, and gestational diabetes^**(18)**^, as illustrated in Figures 3 to 6.

The rate of prenatal diagnosis of esophageal atresia ranges from 16% to 37%, with most cases being confirmed postnatally. In suspected cases, a nasogastric tube (8 or 10 French) is inserted in the delivery room. The tube is measured from the external auditory meatus to the mouth, plus the distance from the mouth to the contralateral costal margin. Failure of the tube to advance to the expected length (typically 9–12 cm) support the suspicion of esophageal atresia. Even when prenatal imaging and physical examination strongly suggest the diagnosis, postnatal imaging remains an option for confirmation^**(19,^20^)**^. These aspects are explored in further detail in subsequent sections and summarized in Chart 2.

**Chart 2:** —Summary of major esophageal and gastric anomalies: imaging features, differential diagnosis, and management considerations.

Anomaly	Prenatal imaging findings	Key imaging sign	Main differential diagnoses	Management implications
Esophageal atresia (with or without tracheoesophageal fistula)	Small/absent stomach bubble; polyhydramnios; pouch sign on MRI	Pouch sign	Duodenal atresia; diaphragmatic hernia; congenital infection	Delivery planning at a tertiary center; postnatal surgical repair
Congenital esophageal stenosis	Normal stomach bubble; mild polyhydramnios	Segmental narrowing (postnatal imaging)	Webs; vascular rings; external compression	Postnatal endoscopic/ surgical correction
Hypertrophic pyloric stenosis	Dilated stomach; thickened pyloric muscle postnatally	Target sign (thickened pyloric muscle)	Antral stenosis; antral diaphragm	Surgical pyloromyotomy
Antral atresia/stenosis	Dilated stomach with no distal passage; polyhydramnios	Dilated stomach with no distal passage	Duodenal atresia; annular pancreas	Early postnatal surgery; antropyloroplasty
Short esophagus/ hiatal hernia	Thoracic stomach position; abnormal cardiac-axis displacement	Thoracic stomach position	Diaphragmatic hernia	Requires multidisciplinary evaluation and surgical correction

The etiology of esophageal atresia remains unclear, and no definitive genetic predisposition has been identified. However, studies suggest an association between esophageal atresia and trisomies 18 and 21^**(21)**^. Approximately 19% of newborns with esophageal atresia meet the criteria for the vertebral defects, anal atresia, cardiac defects, tracheoesophageal fistula/esophageal atresia, renal anomalies, radial aplasia, and limb abnormalities (VACTERL) association^**(22)**^. Recognition of the VACTERL association is crucial because it directly affects prognosis and management.

Ultrasound remains the first-line modality for prenatal evaluation because of its accessibility, dynamic imaging capabilities, and safety profile^**(23,^24^)**^. However, its diagnostic accuracy is highly dependent on operator expertise and gestational age, with reported sensitivities for esophageal atresia ranging from 40% to 60%. Common pitfalls include transient gastric filling, incomplete fetal swallowing, and motion or acoustic artifacts, which may mimic or obscure true anomalies. The use of MRI offers superior soft-tissue contrast and spatial resolution, with sensitivity and specificity rates up to 80–90% for gastrointestinal malformations. It is particularly valuable when ultrasound findings are inconclusive, although fetal movement and technical limitations may still affect image quality. A combined approach often optimizes diagnostic confidence and guides perinatal management.

Emerging technologies such as three-dimensional (3D) and 4D ultrasound, diffusion-weighted MRI, and artificial intelligence-assisted segmentation are increasingly being integrated into prenatal imaging protocols. These advances enable enhanced visualization of fetal anatomy, more precise volumetric assessments, and improved detection of subtle anomalies, thereby supporting more accurate diagnoses and individualized treatment planning.

##### Prognosis

After diagnosing esophageal atresia, further evaluation is necessary to identify associated abnormalities, including cardiac, gastrointestinal, genitourinary, skeletal, and neural malformations such as meningocele or hydrocephalus. In the absence of additional anomalies, the prognosis is favorable, with a survival rate of approximately 90%, regardless of the presence of a tracheoesophageal fistula. Advances in anesthetic and perinatal care have significantly reduced morbidity and mortality in patients with esophageal atresia^**(14)**^.

The prognosis primarily depends on the presence of congenital defects, gestational age, birth weight, respiratory complications (e.g., infections), and cardiovascular issues. Outcomes are better when birth weight exceeds 2,500 g and there are no respiratory complications or associated cardiac anomalies. Therefore, mortality and adverse outcomes in newborns with esophageal atresia are more influenced by these factors than by the type of atresia or the presence of a fistula. To refine the prognosis and optimize individualized treatment, preoperative risk classifications have been developed. The Waterson classification categorizes newborns into three groups^**(25)**^: Group A includes those weighing over 2,500 g without complications, with a survival rate exceeding 95%; Group B includes those weighing 1,800–2,500 g without complications or with moderate pneumonia or a congenital anomaly, with a survival rate of 50–60%; and Group C includes those weighing under 1,800 g or over 2,500 g with pneumonia or severe congenital anomalies, with a survival rate of only 10–20%. A more recent and more widely used model is the Spitz classification^**(22)**^, which stratifies neonates based on birth weight and congenital heart disease^**(26)**^: Group I includes those with a birth weight ≥ 1,500 g without congenital heart disease, with a survival rate of 91–99%; Group II includes those with a birth weight under 1,500 g or with congenital heart disease, with a survival rate of 59–82%; and Group III consists of those with a birth weight under 1,500 g and with congenital heart disease, with significantly lower survival rates (0–50%). Although these classifications continue to be widely used, their limitations should be considered in light of recent advances in neonatal intensive care, particularly for preterm infants, those small for gestational age, and those with associated anomalies.

##### Prenatal management

Recognizing structural abnormalities during prenatal care is crucial for effective decision-making. When esophageal atresia is suspected or diagnosed, the first step is a detailed ultrasound evaluation of the digestive, cardiovascular, pulmonary, and nervous systems^**(27)**^. After diagnosis, serial ultrasound monitoring is necessary to assess and quantify amniotic fluid volume. In severe cases of polyhydramnios, drainage of amniotic fluid may be considered as a management option^**(28)**^. There are no specific contraindications or recommendations regarding delivery in cases diagnosed prenatally. The timing and mode of delivery continue to be determined at the discretion of the attending obstetrician^**(29)**^.

##### Postnatal management

The initial perinatal care includes identifying newborns with signs of esophageal atresia, such as hypersalivation, through clinical examination. If suspected, a nasogastric tube should be inserted^**(27)**^. Chest X-ray is the preferred diagnostic method, and contrast-enhanced studies are contraindicated because of the risk of bronchial aspiration.

**Figure 7:**
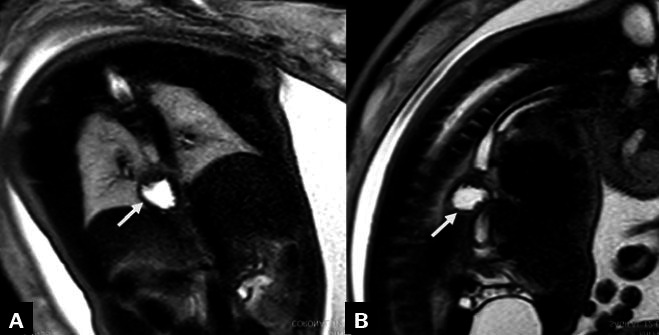
Two-dimensional ultrasound images, acquired with a convex 3.5 MHz transducer in the coronal and sagittal planes (**A** and, **B**, respectively), of a fetus with a hiatal hernia (arrows) at 30 weeks of gestation. Prenatal diagnosis of a hiatal hernia is rare, and the condition may be misinterpreted as any one of a number of other cystic thoracic anomalies.

Preventive measures should be taken immediately to reduce aspiration risks, even before the diagnosis has been confirmed. Newborns with esophageal atresia cannot swallow or pass fluids through the gastrointestinal tract, leading to hypersalivation and increased aspiration risk. If a tracheoesophageal fistula is present, the abnormal communication between the digestive and respiratory tracts further heightens that risk^**(30)**^.

Newborns should be positioned at a 45-degree incline with a nasogastric tube in the proximal esophageal pouch under continuous suction to prevent bronchial aspiration and pneumonia, a critical prognostic factor. These measures should continue until surgical correction. Parenteral nutrition should be initiated to support weight gain and optimize surgical outcomes, reducing the urgency of intervention^**(27)**^.

Surgical correction depends on esophageal length, which may limit primary anastomosis. In Gross type C atresia (with a distal fistula), repair is typically immediate, with intraoperative assessment determining feasibility. In pure atresia (without a fistula), the distance between segments is measured; if it exceeds two vertebral bodies, esophagostomy/gastrostomy and fistula correction are initially performed, followed by esophageal substitution surgery. Esophageal elongation techniques, such as the Kimura and Foker procedures, may facilitate primary anastomosis^**(31)**^.

Surgical complications include anastomotic stricture, recurrent tracheoesophageal fistula, leakage, dehiscence, and gastroesophageal reflux. Postoperatively, 85% of infants experience reduced distal esophageal motility, with reflux occurring in up to 50%. Acid-suppressive therapy is recommended for reflux management^**(17)**^.

#### Less prevalent esophageal malformations

In addition to fistula and atresia, the esophageal lumen may exhibit narrowing, known as esophageal stenosis. This condition results from decreased or nonexistent blood flow, incomplete recanalization, or vascular malformations, typically affecting the distal third of the esophagus. Stenosis is often linked to incomplete esophageal development in the eighth week of embryogenesis, leading to insufficient vascularization and subsequent muscular atrophy of the esophageal wall^**(32)**^.

**Figure 8:**
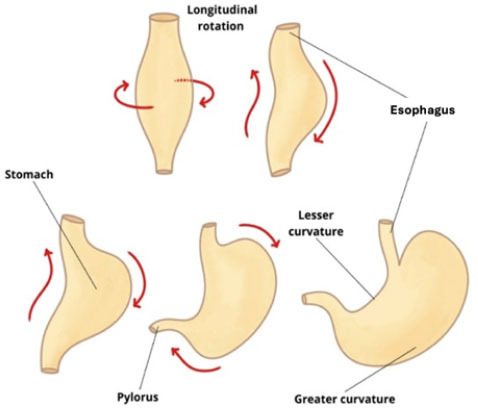
Schematic illustration of embryonic stomach development and rotation, created for didactic purposes.

Another malformation, short esophagus (or congenital hiatal hernia), arises from incomplete elongation of the esophageal tube, causing the stomach to migrate toward the esophageal hiatus and potentially into the thoracic cavity. The exact pathophysiology remains unclear^**(33)**^. Due to its rarity^**(^34^)**^, morbidity and mortality rates are not well established. The diagnosis relies on imaging, which reveals displacement of the stomach into the thorax instead of the abdomen (Figure 8).

### Stomach

#### Developmental aspects

The stomach develops in the fourth week of embryogenesis from a fusiform dilation of the cephalic portion of the foregut. Over the following weeks, differential growth rates in gastric regions cause a morphological and positional transformation. The stomach rotates approximately 90 degrees along its longitudinal axis, shifting the left side anteriorly and the right side posteriorly. This rotation positions the left vagus nerve on the anterior wall and the right vagus nerve on the posterior wall. In addition, the posterior wall expands more than the anterior, forming the greater and lesser curvatures^**(35)**^.

As the stomach grows, the dorsal mesogastrium shifts leftward, forming the omental bursa along the longitudinal axis. While the cephalic and caudal ends initially remain in the midline, a second rotation occurs along the anteroposterior axis. This movement repositions the pyloric (caudal) portion upward and to the right, while the cardiac (cephalic) portion moves downward and to the left^**(9)**^. The embryonic repositioning of the stomach is illustrated in Figure 8.

The embryological rotation of the stomach, which positions the fundus anteriorly and to the left, is directly reflected in fetal imaging. On prenatal MRI and advanced ultrasound, this anatomical orientation allows for accurate identification of the gastric fundus and facilitates differentiation between normal and abnormal positioning.

Understanding these developmental processes is crucial for interpreting images of the fetal digestive tract and recognizing malformations.

Stability is ensured by ligament formation. The falciform ligament and lesser omentum arise from the rightward displacement of the ventral mesogastrium. The greater omentum forms through mesodermal proliferation in the dorsal mesogastrium, and the gastrolienal ligament connects the stomach to the spleen^**(8)**^. Developmental failures during these processes can lead to fetal gastric malformations, as discussed in the following sections.

#### Antral stenosis

##### Definition

Antral atresia, also known as congenital pyloric atresia, is a rare upper digestive tract malformation characterized by complete obstruction or absence of the pyloric canal. Antral stenosis, similarly, involves narrowing of the lower stomach, restricting gastric content passage into the duodenum. This condition may result from congenital anomalies such as antral membranes or diaphragms, leading to partial gastric outlet obstruction^**(36,^37^)**^. Both conditions present similar symptoms and require early diagnosis and appropriate intervention.

##### Embryology

Antral stenosis manifests as gastric outlet obstruction, often due to an antral diaphragm—a membranous structure in the prepyloric region causing partial obstruction. This condition primarily affects infants and children, typically presenting as non-bilious vomiting shortly after birth or in early childhood. Although its exact etiology remains unclear, it is believed to stem from excessive localized endodermal growth during early gastric development^**(38)**^.

Stomach development is a highly regulated process involving multiple signaling pathways. Fibroblast growth factor 10 and its receptor (fibroblast growth factor recep-

tor 2b) are essential for normal gastric morphogenesis. Murine studies have indicated that the absence of these factors results in smaller stomachs with reduced glandular complexity and abnormal epithelial differentiation^**(39)**^. Disruptions in these signaling pathways may contribute to developmental anomalies such as antral stenosis.

##### Clinical manifestations

Antral stenosis commonly presents with non-bilious vomiting, which may be projectile, and epigastric pain. Neonates typically show signs of gastric outlet obstruction, including vomiting and failure to thrive, often within the first days of life. Antral stenosis is often accompanied by prenatal polyhydramnios^**(36,^39^)**^. Older children may experience a postprandial sensation of fullness, as well as belching^**(36,^39^)**^. In some cases, antral stenosis mimics hypertrophic pyloric stenosis, particularly when distal antral hypertrophy is present^**(39)**^. Symptom severity depends on the degree of stenosis or atresia, and associated anomalies should always be considered.

##### Diagnosis

Barium meal examinations help identify structural anomalies by assessing gastric transit^**(40)**^. However, prominent mucosal folds may lead to misinterpretation, requiring clinical correlation. Ultrasonography, often the first-line imaging modality because of its accessibility, aids in differentiating antral stenosis from similar conditions. Endoscopy remains the gold standard, providing direct visualization of the gastric antrum, confirming antral diaphragms, and enabling therapeutic intervention when needed^**(41)**^.

##### Treatment

Surgical intervention is the primary treatment for antral stenosis. Procedures include antral diaphragm resection to restore normal gastric emptying and antropyloroplasty to prevent future obstruction. Surgery generally resolves symptoms and restores gastric function, with a low recurrence rate^**(39)**^.

##### Prognosis

When treated early, antral stenosis has a favorable prognosis, with high survival rates and good long-term outcomes. Prognosis depends on birth weight, associated anomalies, and postnatal clinical conditions. Postoperative complications, including anastomotic stenosis and gastroesophageal reflux, may require prolonged follow-up and additional interventions. Early diagnosis and coordinated care among obstetricians, neonatologists, and pediatric surgeons are essential for optimizing outcomes^**(42)**^.

#### Other gastric malformations

Fetal gastric anomalies include pyloric duplication and prepyloric mucosal diaphragm. Pyloric duplication is rare, with a prevalence of only 0.06–0.40%^**(43)**^, and is characterized by a gastroduodenal fistula forming a second canal. Most newborns are asymptomatic, and, in many cases, the diagnosis is not made until complications (prepyloric ulcers, peptic disease, fibrosis, or chronic inflammation) arise^**(44)**^.

**Figure 9:**
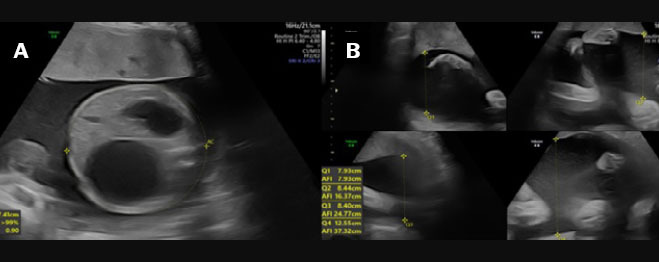
Two-dimensional ultrasound images, acquired with a convex 4–8 MHz transducer in the sagittal and axial planes (**A** and **B**, respectively), of a fetus with duodenal atresia at 28 weeks of gestation, showing the “double-bubble” sign (in **A**) and polyhydramnios (in **B**).

The prepyloric mucosal diaphragm partially obstructs the gastrointestinal tract through a prepyloric membrane. Its clinical presentation resembles stenosis but is less severe, because complete obstruction does not occur. Albeit more insidious, it can still lead to polyhydramnios, postprandial vomiting, and abdominal distension^**(45,^46^)**^. Symptoms vary depending on the severity of the obstruction and can mimic food intolerance, gastroesophageal reflux, hiatal hernia, intestinal motility disorders, or other congenital obstructive pathologies^**(47)**^, such as duodenal atresia (Figure 9).

## CONCLUSION

Fetal esophageal and gastric anomalies demand an integrated diagnostic and therapeutic approach. For radiologists, adherence to standardized prenatal imaging protocols is essential—beginning with detailed secondtrimester 2D and 3D/4D ultrasound to assess the stomach bubble, esophageal pouch, and amniotic fluid volume. A referral for MRI should be considered when ultrasound findings are inconclusive, when there is suspicion of complex anatomy, or in cases of persistent polyhydramnios without a clear etiology, and the examination should ideally be performed between 28 and 32 weeks of gestation for optimal anatomical resolution. Radiologists should maintain proactive communication with obstetric and neonatal teams, promptly reporting critical findings and participating in multidisciplinary case discussions to guide delivery planning and postnatal management. This collaborative approach, supported by protocol-driven imaging and timely referral, enhances diagnostic accuracy, facilitates early intervention, and optimizes neonatal outcomes. Continued technological advances and registry-based epidemiological studies remain vital to further refine prenatal diagnosis and improve the long-term prognosis.

## Data Availability

Not applicable
